# Navigating oneself through the eyes of the other – meanings of encountering ambulance clinicians while being in a suicidal process

**DOI:** 10.1080/17482631.2024.2374751

**Published:** 2024-07-02

**Authors:** Staffan Hammarbäck, Lena Wiklund Gustin, Anders Bremer, Mats Holmberg

**Affiliations:** a Faculty of Health and Life Sciences, Linnaeus University, Växjö, Sweden; b Centre of Interprofessional Collaboration Within Emergency Care (CICE), Linnaeus University, Växjö, Sweden; c Department of Ambulance Service, Region Sörmland, Katrineholm, Sweden; d Centre for Clinical Research Sörmland, Uppsala University, Eskilstuna, Sweden; e School of Health, Care and Social Welfare, Mälardalen University, Eskilstuna/Västerås, Sweden; f Department of Health and Care Sciences, UiT/The Arctic University of Norway, Narvik, Norway

**Keywords:** Ambulance clinicians, lived experience, phenomenological hermeneutical, suicidal process, suicide prevention

## Abstract

**Purpose:**

The suicidal process contains both observable and non-observable phases, and patients have described the process as characterized by loneliness and darkness. Ambulance clinicians encounter patients in all phases of the suicidal process but little is known on what meaning this encounter has to the patients. The aim of this study was to elucidate meanings of encountering ambulance clinicians while being in a suicidal process.

**Methods:**

Data were collected through fifteen individual interviews with eight participants who had lived experiences of encountering ambulance clinicians. Inductive design using phenomenological hermeneutical approach was used.

**Findings:**

Patients are impacted by the clinicians, both in how they find their value in the situation, but also in expected trajectory. Three themes; ‘Being impacted by representatives of society’, ‘Being unsure of one´s own value’ and ‘Regaining hope in moments of togetherness’ generated the main theme ´Navigating oneself through the eyes of the other´.

**Conclusion:**

The way ambulance clinicians communicate impacts how patients navigate themselves in the ambivalence about living or dying, and the encounter either consolidate a feeling of being a burden, or instil hope of an endurable life. Through conversation, clinicians could support the patients in taking the first steps in the journey of recovery.

## Background

Every year, more than 700.000 people die in the world due to suicide and it is estimated that for every suicide there are up to 20 suicide attempts (World Health Organization, 2021). A suicide is preceded by a suicidal process, where feelings and suicidal ideation vary in intensity (Wasserman, 2016). The characteristics and the duration of the process are individual, and commonly the process stretches over months or years. The suicidal process includes feelings of weariness for life, suicidal ideation, suicidal behaviour and suicide. However, most people with suicidal ideation do not die from suicide. Suicidal ideation is defined as thoughts about self-harm, with deliberate consideration or planning of possible techniques of causing one’s own death (American Psychiatric Association, 2013). Suicidal behaviour is understood as any behaviour, with underlying suicidal ideation, that has some intent of causing one’s own death (Goodfellow et al., 2018). It can be hard to distinguish between suicidal behaviour and deliberate self-harm without suicidal intention, and many times patients assess their suicidal intent higher than clinicians (Hatcher & Pimentel, 2013). There are several risk factors for suicide at individual, social and environmental levels (Turecki & Brent, 2016). Individual risk factors include physical and mental illness, with the greatest risk factor being previous suicide attempts, self-harm and depression. Social risk factors are for instance social isolation and financial problems, while environmental risk factors include access to means or poor access to mental health care.

The suicidal process consists of both observable and non-observable phases and the process commonly stretches over months or years but can be as short as hours or minutes in exceptional circumstances (Wasserman, 2016). Being in the process has been described as being alone in darkness which later turned into an unbearable situation (Vatne & Nåden, 2012). Persons also describe longing for someone who would be able to see, listen and understand. Direct communication about suicidal ideation can be difficult, and there is a fear of making the situation worse, stigmatization, feelings of invalidation or being a burden to others (Krychiw & Ward-Ciesielski, 2019). There is an ambivalence and a struggle between life and death, between longing for life and a desire for relief from suffering. It is usually not death in itself that is the goal but to get away from the perceived endless suffering (Pavulans et al., 2012). When perceiving to be unable to handle various sorts of life problems, having the option of suicide can entail a sense of control in a situation characterized by lack of control. Persons who attempted suicide also describe feelings of failure, distress and shame. However, these feelings are alleviated and made endurable when being treated with respect and kindness (Wiklander et al., 2003). Hope can arise when there is a feeling of being understood and listened to (Vatne & Nåden, 2018). This can also create feelings of hope that there is still an ability to influence one’s own life situation. Beginning to talk about the suffering can decrease loneliness and give hope of establishing contact with others. After a suicide attempt, difficulties in life are not changed as there can still be feelings of loneliness and guilt. However, these feelings can be alleviated when clinicians instil a feeling of being valuable as a human being who has potential in life. The clinicians’ attitude and the atmosphere of care can contribute to patients’ recovery.

Internationally, it differs in terms of which professions work in the ambulance service, but usually ambulance crews consist of paramedics, emergency medical technicians or physicians (von Vopelius-Feldt & Benger, 2014). In Sweden, the profession consists of at least one registered nurse with or without specialization (Sjölin et al., 2019). In assignments that involve patient suicide risk, there is often collaboration with the police service (Nilsson et al., 2017; Soares & Pinto da Costa, 2019).

Ambulance service work is mainly based on guidelines and routines, although ambulance clinicians also personalize and make individual adjustments to their way of work (Corman, 2018). Required skills cover scene management and medical, technical, communicational and becalming care which includes body language and sharing of information to the patient (Kyed, 2020). Even though most causes of contact with the ambulance service are due to physical problems, patients with mental illness, including self-harm and suicidal ideation, are frequently present in ambulance care (Rees et al., 2018). Of those who die within a year from contact with the ambulance service due to mental illness, suicide has been reported as the most common cause (Duncan et al., 2019). In ambulance care, patients’ medical needs tend to be prioritized over their mental needs (Rees et al., 2015). Furthermore, the patient relationship tends to get less focused in challenging and critical situations (Svensson et al., 2019). Encountering patients who self-harm can be challenging for ambulance clinicians as they perceive having a lack of competence in assessing and treating these patients (Zetterberg et al., 2022). Patients assessed by ambulance clinicians as seeking help due to mental illness are more likely to be non-conveyed (Lederman et al., 2020). Additionally, nurses in emergency care have been reported as having problematic attitudes towards suicidal behaviour, including apathy and considering suicidal patients as not being seriously ill (Bolster et al., 2015). Patients who encountered hospital emergency care following self-harm experienced both hostility and gentleness (MacDonald et al., 2020). For instance, patients described being punished and receiving treatment that made them less likely to engage in the future help-seeking or disclosure. However, there were also experiences of being treated with respect and consideration, and where the positive relationship was understood as part of the recovery.

Patients in suicidal processes describe vulnerability and ambivalence about living, and how encountering clinicians impacts on future help seeking and recovery. Encountering ambulance clinicians can take place in crucial moments, for instance after suicide attempts, yet little is known on what meaning this encounter has to the patients. Therefore, the aim of this study was to elucidate meanings of encountering ambulance clinicians while being in a suicidal process.

## Theoretical perspective

From a caring science perspective, suicidality can be understood as overwhelming suffering. According to Fredriksson (2003), caring conversations can alleviate suffering and hold three aspects—the relational, the narrative, and the ethical. The relational aspect is characterized by presence, touch and listening (Fredriksson, 1999). Presence is either “being there” or “being with”. “being there” encompasses communication and understanding and is understood as an answer to the patient’s need, commonly described as an action with the purpose of achieving a goal. “being with” is when the nurse enters the patient’s world, remains with the patient, endures feelings of discomfort and offers comfort. Touch is understood as the way of creating and maintaining connection with the patient and is dependent on whether the nurse and patient are in tune with each other. Listening includes being silent, not only silence of words but also silence of own thoughts, in order to give the patient space and time for their own interpretation of the experience. The narrative aspect of the caring conversation can generate understanding and meaning of the experience (Fredriksson, 2003). However, suffering can temporarily silence a patient’s voice due to fear of not being heard or causing other people harm. Therefore, the patient needs someone who can listen receptively. The ethical aspect is based on the initial asymmetry of power in the relationship due to the patient’s suffering. Through mutual respect, the asymmetry can be balanced and help the patient to restore autonomy and power. Words and language become the medium through which events and actions can be valued against life as a whole. The Tidal Model (Barker & Buchanan-Barker, 2005) contributes further to the understanding of how caring conversations and relationships with clinicians can be part of the patient’s journey of recovery. Recovery can be understood as a way of reclaiming one’s story and finding resources within oneself to undertake the journey in the inevitable change that is life.

## Research design

An inductive, phenomenological hermeneutical approach (Lindseth & Norberg, 2004, 2022) was used to elucidate essential meanings of the phenomenon *encountering ambulance clinicians while being in a suicidal process*. The design is grounded in the phenomenology of Husserl (2014) and the hermeneutics of Ricœur (1976, 1991). The methodology relies on the dialectics of understanding and explaining. According to Ricœur (1991), a transcription of an interview should be considered as a text, which has the potential to open up a world of its own in front of the text. In other words, it is the text as text that is in focus for analysis and interpretation, not the person. Hence, hermeneutic interpretations involve both searched explanations of the text structure and understanding of the meaning of the text. However, the explanations on which hermeneutical interpretations rely are not causal. Rather, the focus is on how the text is constructed, which will be further described below. The focus on the structure of the text also creates a distance to one’s preunderstandings, while the following interpretive step (see below) contributes to a deeper, hermeneutic understanding. While the former is a matter of deconstructing the text, the latter is about reconstructing a new understanding of the lifeworld phenomenon, which unravels through narratives of lived experiences.

### Participants and research context

A purposeful sampling was used to recruit participants from psychiatric outpatient admission clinics in one region of Sweden. Psychiatric clinicians identified persons who had been in contact with ambulance care in the last year while being in a suicidal process and asked if the researchers were allowed to contact them to provide further information about the study. Contact details from 12 persons were handed over to the researchers. There were four dropouts; one person did not respond to the researchers’ contact attempts, two persons whom at first accepted participation did not respond when the time of the interview was to be decided, and one person with whom the interview was interrupted as the person had very scarce memories from encountering the ambulance clinicians. Interviews were made with the remaining eight persons, of which four were women and four were men. The age of the participants ranged from 25 to 55 (mean 33).

### Data collection

In order to understand the participants’ lifeworlds, individual narrative interviews (Brinkmann & Kvale, 2015) were conducted between May 2021 and May 2022. The first participant offered to be contacted again, and after a discussion among the authors, this offer was accepted, and a follow-up interview was conducted (Read, 2018). The follow-up interview enabled the possibility to go deeper into the meanings of the encounter and, therefore, the following participants were asked for a follow-up interview after the first interview. Follow-up interviews were made between 4 and 20 days after the first interview. One person declined the follow-up interview without having to state any reason. In total 15 interviews were conducted according to participant preference; either by telephone (*n* = 6), in the participants' home (*n* = 2) or in a room provided by the healthcare region (*n* = 7). The first round of interviews started with the open-ended question “*Can you tell me about your encounter with the ambulance care*”? Follow-up interviews started with the question “*Have you thought about anything regarding the encounter with ambulance clinicians, since the first interview*”? The first round of interviews lasted between 27 and 53 min (mean 36 min) and the follow-up interviews lasted between 23 and 44 min (mean 33 min). All interviews were audio recorded and transcribed verbatim by the first author.

### Data analysis

A phenomenological hermeneutical method based on Ricœur’s thinking was used to analyse the data (Lindseth & Norberg, 2004, 2022). The analysis comprised three phases. The first phase was the *naïve reading*, in which the transcripts were read through several times, leading to a naïve understanding of the data as a whole. This understanding needs, however, to be challenged. Hence, in the second phase, the *structural analysis* focused on how the text is structured around different, reoccurring themes. First, meaning units were identified in the text, and the meanings were condensed. The condensations were compared and stratified into subthemes, later grouped under themes from which the main theme could be generated. Examples of the structural analysis are presented in Table I. This explanatory phase created a distance to the preunderstandings that might be reflected in the naïve understanding, as it de-contextualize the narratives that are reflected in the text as a whole. Following Lindseth and Norberg (2004), this can validate the naïve interpretation, generating a dialectic movement between understanding (i.e., naïve interpretation) and explanation (i.e., structural analysis) (Ricœur, 1991). As described by Ricœur (1988, 1991), this can also give rise to seemingly different and even paradoxical and conflicting interpretations. However, such differences can be united in what Ricœur describes as heterogeneous synthesis that adds further nuances to the understanding of the complexity of human experiences. Therefore, in the third phase of the analysis, the *comprehensive understanding*, the naïve understanding and the structural analysis were interpreted in the light of each other and the theoretical perspective to obtain a deeper understanding of the phenomenon. After the structural analysis, there was a discussion between the authors on which theory the findings could be interpreted against. The Tidal Model (Barker & Buchanan-Barker, 2005) was found suitable with its idea of how a persons’ sense of self is inextricably tied to the life story and the meanings generated within the story. Furthermore, Fredriksson’s (2003) theory of caring conversations seemed to complement The Tidal Model and deepened the comprehensive understanding.

**Table I.** Examples from the structural analysis.

Meaning unit	Condensation	Subtheme	Theme
*They definitely had power over, I mean my prejudice so to speak, and how they would be affected. (…) When I say power, I mean how they impact on how I think and feel about myself after the encounter. In their position, they affect me, which is what I mean by power.*	Ambulance clinicians have power over self-image.	Being inferior to authority	Being impacted by representatives of Society
*I want to tell them what has happened, what I have taken, and can you contact this person and I want to know what is going on (silence) I do not know, just to be seen as a human being, and not just like a medical project that is lying here (silence).*	Not being given an opportunity to speak is being unseen as a human being and regarded as a medical project.	Being a burden	Being unsure of one´s own value
*What you want the most when you feel so bad is to not feel lonely. I have always said that loneliness is a feeling, it is not a fact! You can feel lonely even when you are surrounded by thousands of people. You just want someone who sees and listens, and if you do not have that, you feel the loneliest. (…) They can decrease the feeling of loneliness when they talk, I mean with the right tone and truly trying to see the person.*	Being lonely in the process. A longing not to be alone. In the encounter loneliness decreases when being seen and listened to.	Re-entering human community	Regaining hope in moments of togetherness

### Ethical considerations

The study was approved by the Swedish Ethical Review Authority (No 2019–03774 and 2021–00078) and performed in accordance with the Declaration of Helsinki. Concerning the ethics of research benefits and harm, the possible stressfulness that the interview situation could cause the participants was weighed against the important knowledge that could come from the study. Holloway and Freshwater (2007) argue that there are several reasons for including vulnerable person into research interviews. Narrating can be seen as a way for participants to regain power and control. It can also help to make sense of the past from the perspective of the present. Sharing experiences has also been described as meaningful from an altruistic point of view, where participants describe a desire to help others in similar situations (Dyregrov et al., 2010). In this study, psychiatric clinicians were instructed to identify persons who understood what it meant to participate in an interview study and were assessed to be able to manage an interview situation. The question of participation was asked by the first author, after providing verbal and written information about the study and that participation was voluntary and that they could withdraw their participation without stating any reason. The participants signed a written consent of participation. Before conducting the interviews, the participants’ social network was looked over to make sure there was available support if the interview situation became overwhelming. The regional division manager of psychiatric care signed a document that declared that the region had sufficient resources to support participants, if the need arose. After the interview ended, the participants were asked whether they felt the need for further support. The participants’ names were anonymized and replaced with codes during the analysis. The code key is only accessible to the first author.

## Findings

### Naïve understanding

Encountering ambulance clinicians, while being in a suicidal process, means encountering representatives of the human community where the ambulance service symbolizes security and comfort. At the same time, experiencing oneself as a burden means that the clinicians would rather attend someone who deserves care. Being in a suicidal process is painful, lonely and to be secluded from the human community. It is to be exposed and vulnerable, and the clinicians have impact on the view of oneself. Distressful feelings are reduced and hope of an endurable life arises when the clinicians engage in conversations and show that they try to understand. However, trusting the clinicians is a step-by-step process, and the conversation is the foundation of building a relationship. There is a sensitivity to the clinicians’ responsiveness in the conversation and their ability to listen. Being listened to is having value as a human being and thus the clinicians have the power to show that re-entering the human community is possible. Seeing oneself as a problem is consolidated when the clinicians are without empathy and detached from feelings. This influences trust and expectations in other parts of the healthcare system.

## Structural analysis

The structural analysis resulted in the main theme ´*Navigating oneself through the eyes of the other*, comprised of three themes and eight subthemes (see Table II).

**Table II.** Main theme, themes and subthemes.

Main theme	Navigating oneself through the eyes of the other		
Themes	Being impacted by representatives of society	Being unsure of one´s own value	Regaining hope in moments of togetherness
Subthemes	Being inferior to authority	Keeping up a façade	Re-entering human community
	Being ambivalent in the presence of life preservers	Being a burden	Having one’s story visualized through the relationship
	Being a patient or a criminal		Being assembled from pieces

## Navigating oneself through the eyes of the other

To navigate includes both position and expected direction. It means being inferior and unsure of own value, and at the same time the encounter sets expectation of what is to come. To navigate oneself means being impacted by the ambulance clinicians who represent society and human community and there is a sensitivity to the clinicians’ way of communicating. There is a paradox in the encounter as the ambulance clinicians are seen as preservers of life while being ambivalent in the struggle between life and death. In the ambivalence about living or dying, there is uncertainty about having human value. The encounter can consolidate being a burden if the ambulance clinicians show no interest in oneself as a unique human. However, the encounter can also give hope of an endurable life, if clinicians give an invitation to a relationship in which they are compassionate, listening and trying to understand.

### Being impacted by representatives of society

Ambulance clinicians are representatives of society and to encounter them is to encounter authority and to be inferior. To get help is to subordinate, but there is also doubt that help is available. In the ambivalence about living or dying, a paradox occurs when encountering preservers of life, as life in itself is a problem. Vulnerability is increased when police officers are present, and it changes the dynamics in the encounter with ambulance clinicians and implies being criminal.

#### Being inferior to authority

The entrance to the encounter is a power-imbalance where the ambulance clinicians, as representatives of society, are the stronger part. To be inferior means to subordinate if help is to be received, as the clinicians are the first in line to the healthcare system. It is in the clinicians’ power to gate keep and set an expectation of how other clinicians in the healthcare system will understand the situation. To subordinate is to follow the clinicians’ routines and to let them do their work by conducting medical assessments. The inferior position means proving you are someone in need of help or having to behave properly. At the same time, there is doubt that the clinicians can help or guide to help when nothing has helped before. The power imbalance is elucidated through the clinicians’ body language and the tone in their voice. Being inferior is prominent when clinicians are standing above or are using a cold tone in their voice, which increases vulnerability and exposure. The clinicians’ position of power is further highlighted when the conversation only focusses on diagnosis or suicidal acts, instead of underlying feelings and distress, or if the clinicians appear to be detached from feelings.

They are like robots you know, they come there, and they are going to do their job and walk away. There is no warmth in how they talk, and I find it very hard to respond when I cannot really read them. (Participant 5)

On the other hand, the power imbalance is decreased when the clinicians are personal and show empathy which provides a chance to co-operate and instils control of the situation.

#### Ambivalence in encountering preservers of life

Being ambivalent is to struggle between life and death. There is a desire for a different life that does not have to end with suicide, but in the situation that future seems to be out of reach. Life appears unendurable and death seems to be a way out of the misery. Therefore, going along with the ambulance clinicians is to trust them that there is hope of an endurable life, not only a matter of survival.

When you feel like that, you are not even sure that you want to go on living, you sort of put your life in their hands when you trust them and step away from the railway. (Participant 6)

Encountering the clinicians is a paradox. It is to encounter those who are put to preserve life while being ambivalent in the struggle between life and death. The ambulance symbolizes safety and help, but there is an uncertainty if help is possible. The paradox is further elucidated when the clinicians as preservers of life, through condescending and judgemental language, reinforce the idea of death as the way out of misery. After a suicide attempt, the encounter brings contradictory feelings; there is disappointment that one has survived. There is the pain and anger after the realization that the attempt was hindered, but also hope that there is help available and a will to live can arise.

There is this ambivalence somehow. In one way I do not want to go along with them at all, but in another way, you have this bloody survival instinct that kicks in. (Participant 7)

The encounter induces shame of occupying resources from those who are experienced to deserve care and who want to live. At the same time, it is stressful and can activate an instinct of flight as the clinicians’ arrival could restrict one’s possibilities to act.

#### Being a patient or a criminal

The dynamics in the encounter with the ambulance clinicians change when police are called to the scene along with the ambulance. The presence of police officers increases vulnerability and can make it harder to open up and speak freely about oneself and the situation. As there is a fear of being seen as a criminal, consumption of drugs might not be shared to the ambulance clinicians in the presence of police officers. The seriousness of the situation is acknowledged when several authorities are called for, but at the same time, feelings of being a criminal or a mad person arise.

If you go away in a police car, it means that you are a criminal. While if you go in an ambulance, you are a patient. That is the difference, and you would rather not look like a criminal. (Participant 2)

The police service is a different authority to the ambulance service and is more controlling, while the ambulance service represents help by offering care. The presence of police officers means being something inconvenient to society or having done something wrong.

### Being unsure of one´s own value

Being in the suicidal process is to doubt one’s own value and to experience oneself as a burden. There is fear of being judged for being in a situation that requires contact with ambulance care. This is accentuated when the clinicians’ communication reveals if one is now a burden to them as well. Trusting them depends on their responsiveness in the conversation and their ability to strive for understanding. The experience of being a burden decreases if the clinicians initiate a conversation, in which there is a possibility to talk about suicidal ideation and feelings that are hidden behind the façade.

#### Being a burden

Being in the situation that requires contact with the ambulance service means being a failure. The responsibility is experienced as one’s own for getting in the situation and this produces a fear of being a burden to the clinicians, as well to oneself. When clinicians show irritation or appear uninterested, it acknowledges one as a problem to them, further elucidated in their use of impeaching language, like *why* or *again*. It means being without human value if clinicians look away, being silent or only talking to each other and this also implies that the clinicians would rather have attended someone else.

You feel like dirt on the floor, only like a problem and you do not follow what is going on. You feel invisible, and you sort of already do that when you want to take your life. You feel like a problem (…) it felt very cold of them not to talk to me. (Participant 4)

Being a burden is elucidated when medical needs are the sole focus in the encounter. It means being without interest as a human being and being silenced.

They only focus on one thing, the body. The soul they leave for someone else (…) they focus on the body and what is there. They cannot focus on the darkness. (Participant 8)

Being a medical project means placing further load on the healthcare system and being something inconvenient to the clinicians. Conversely, when clinicians show that they are trying to understand the situation and share feelings like frustration, seeing oneself as a burden is alleviated.

#### Keeping up a façade

Being vulnerable in the encounter involves a struggle to protect oneself from being judged, by keeping up a façade. Emotions and personal thoughts are hidden behind this façade as there is uncertainty as to how the clinicians will react, or if it could cause the clinicians distress.

Right when I started talking about it, I felt stupid. So I did not dare say anything more. Yeah, you sort of let some information out and see what you get back. To see if you dare talk more about it or not. (Participant 4)

The façade is used to protect the clinicians as well, because even if talking about suicidal ideation is not hard in itself, there is concern that it could be painful for the clinicians to listen. There is also an understanding that clinicians might have to be distanced and that they do not have interest to further explore suicidal ideation.

I would not think it was particularly fun if I was an ambulance carer, to see people on the brink of ruin when they have given up everything. So, I guess it is sad for the ambulance carer to see people in these situations. (Participant 1)

There is a sensitivity to the clinicians’ responsiveness. Being the vulnerable part in the encounter means that the clinicians must initiate the conversation. If they show that they can listen attentively, an opportunity arises to open up about suicidal thoughts and to stand out from behind the façade.

### Regaining hope in moments of togetherness

Hope arises in the brief relationship when being seen and valued as a unique person. Loneliness alleviates when being reminded of ordinary human to human behaviour through everyday conversations. An opportunity to share one’s own story arises when the clinicians are emotionally available, and the conversation helps to get perspective and a sense of control in the situation.

#### Re-entering human community

Being in the suicidal process is characterized by loneliness. Even if one is surrounded by friends and family, there is a feeling of being different, secluded from others in society and not fully understood. Loneliness is decreased through everyday conversations with the ambulance clinicians as it is a reminder of ordinary human fellowship. For instance, when the clinicians use humour or ask questions about hobbies, pets or education, it lessens tension and highlights important parts in life.

It can seem weird, you were just about to take your life and then someone asks you how old you are, what you like to do in your free time. But at the same time, it feels like someone cares who I am, and not just my sick me. It can give distraction and I know I have told them about my dog, and they have asked questions because that is something that means very much to me. (Participant 6)

Alleviated loneliness brings hope about being part of human community again, without any expectation that the clinicians are to solve any underlying problems. The encounter with the clinicians does not remove the underlying despair, but being treated as an equal human being means being important and it awakes hope of an endurable future.

#### Having one’s story visualized through the relationship

When clinicians are emotionally available, it means that one has impact on them and thus having value as a human being. The relationship is built up gradually, when clinicians are personal and when they show that they are listening and have time. An understanding and non-judgemental attitude means that one can approach and be supported in the pain. Being given the opportunity to talk about underlying feelings and problems provides a chance to get a new perspective and to be in control, which instils hope of a future in which one can handle life.

It was definitely an important impression because in my drug use, I do not have contact with many people and when I have contact … it can go several weeks without me actually having a conversation with someone. So, this conversation I had with the ambulance personnel, it was my first real deep conversation that I have had with anyone in weeks, so that impression lasted! It was huge! It was valuable! (Participant 3)

Through the conversation, there is an opening to come forth as a unique person with a story to tell. When clinicians show that they are listening, being responsive and trying to understand it means being seen and regaining hope. If they are present and emphatic, silence can be a way of showing respect and an opportunity for conversation opens up. Silence is also respectful when emotions are overwhelming and having a conversation is unfeasible.

#### Being assembled from pieces

Being in a suicidal process is to be broken and fragmented. The pieces of oneself can be put together when clinicians are gentle, friendly and interested. If they show that they have not given up hope, one might dare to believe that this situation is only temporary and that something better is possible.

You are a wreck when they come in. They make you feel like you are in safe hands and get your nose just above the water and they make you feel like, well maybe there is human hope left in this world (…) when they come in they are my rock, they get to pick up my rubble, because I am a wreck when I have done this. (Participant 1)

Remaining eye-contact and a warm tone builds up a friendly relationship in which the clinicians share the situation, and their strength is uplifting. Their warmth and confidence convey and awaken hope that life, after all, could be endurable.

## Comprehensive understanding

In the comprehensive understanding, the findings from the naïve understanding and the structural analysis are interpreted in the light of a theory (Lindseth & Norberg, 2004). The main theme and other themes are italicized for clarity. The main theme, *navigating oneself through the eyes of the other*, can be understood in line with the idea of a narrative identity which is being impacted by interaction and communication with others. According to Ricœur (1988), narrative identity means to articulate who we are through narratives, in which we constantly interpret our lives. Our character is constantly formed and reformed, and personal identity is not fully stable or self-transparent, but rather incoherent or even self-alienated. This could be referred to the Tidal model in which mental health is understood as an ever-changing nature of human experience (Barker & Buchanan-Barker, 2005). Mental illness is understood as problems of living, and central to the Tidal Model is the narrative perspective. The person’s story is reality and, in the encounter, the narrative identity is *being impacted by representatives of society*. Navigating oneself in the encounter with authority sets expectations for the future, and not having a personal conversation with the clinicians means being silenced. This reinforces the narrative of oneself, of being a problem, and someone without power and control. *Being unsure of one´s own value* means that there is uncertainty about who one is and what own resources are available. Through a caring conversation (Fredriksson, 2003), characterized by reciprocity and equality, there is a possibility to come forth as a unique person with human value and capacity to change. The conversation contributes to *regaining hope in moments of togetherness*. It is a way of understanding critical events as resetting the compass of life, and the story that emerges from the encounter with ambulance clinicians adds to the existing story of a person’s life (Barker & Buchanan-Barker, 2005). Re-authoring the story is a way of giving meaning to experiences and of being reminded about the important parts in life. It does not only seek to explain the situation, but it also entails the resources of recovery. Navigating oneself encompasses both value and expected trajectory, and in the encounter, the ambulance clinicians become cast in the patients’ stories. As a person’s sense of self is inextricably tied to the life story and the meanings generated within the story, the encounter could be understood as an opportunity to reclaim one’s story. (Barker & Buchanan-Barker, 2005)

## Discussion

The findings in the present study show how patients navigate themselves through the encounter with ambulance clinicians. Ricœur (1988) claims that identity is not fully stable but constantly interpreted through narratives. A person’s story is reality, and how that story is told is impacted by interactions with clinicians (Barker & Buchanan-Barker, 2005). Hustvedt (2013) points out that suicide can be rational from the point of controlling one’s story, as the future seems to be vanished. The hope of a future that does not end with suicide comes from narrating suffering in a relationship with an attentive listener.

The patients in the present study describe an imbalance of power, and to be the inferior part when encountering ambulance clinicians. Being inferior aggravates vulnerability, especially when clinicians are being cold and seem to be detached from feelings. Nelson et al. (2020) found that attending patients who have made a suicide attempt can impact the ambulance clinicians and be emotionally demanding. These encounters can awake personal feelings of vulnerability and distress, and clinicians report pressure to suppress their emotions. Hammarbäck et al. (2023) also describe how clinicians experience vulnerability when being personal in the relationship with patients in a suicidal process. The clinicians describe that they do not engage in conversations as they perceive that they are not able to solve the patients’ problems and therefore run the risk of causing the patients further harm. Avoiding conversations about suicidality could also be a way of protecting from vulnerability. However, this could be troubling as the findings of the present study suggest that being met by someone who is emotionally disconnected increases the feeling of being a burden. Furthermore, the present findings suggest that there are no expectations on the clinicians to solve any underlying problems but what is important is to encounter someone who is emotionally available as it alleviates experiences of exposure and vulnerability.

In fear of being judged, patients describe holding up a façade, but if the clinicians show that they can listen attentively, an opportunity arises to open up about suicidality and distressful feelings. Similar experiences are described by Ganzini et al. (2013) and Richards et al. (2019) where patients open up about suicidality only if the clinicians appear to be interested, truly listening and with a caring attitude. Fredriksson and Lindström (2002) describe how patients keep up a façade to shelter from suffering, anxiety and insecurity. Through a caring conversation, the patients’ narratives of suffering represent a new interpretation of their life story. If the patients are able to abandon the shelter of the façade, interpreting the suffering enables growth and to find meaning in the suffering. This means to risk the very self, but at the same time, there is an opportunity for reconciliation. According to Fredriksson and Eriksson (2003) caring conversations hold an ethical aspect, where the initial asymmetry of power in the relationship has to be balanced in order to restore the patient’s autonomy and power. When patients are enabled to narrate about their suffering, language becomes the medium of a self-interpretative process in which control and meaning can gradually be regained. However, in nursing, conversations run the risk of being reduced to technique and merely a tool to transmit information.

In the findings of the present study, encountering clinicians who are silent is understood as being a burden and taking up their time. Similar understandings of clinicians’ silence is described by Derblom et al. (2021). However, patients in the present study also describe that silence can be respectful and offers an opportunity to talk if the clinicians are emphatic and present. This accords to Younger (1995) who describes that clinicians’ silence can be respectful and filled with communicative meaning and may well be the most constructive response. Furthermore, Younger emphasizes the expressive art of being fully present to another person as the most demanding aspect of caring. This includes being aware of tone of voice, eye contact, body language and being in tune with the patient’s messages. The findings from the present study also suggest that the common everyday conversation alleviated distress as it became a reminder of ordinary human interaction. This is in line with Berglund et al. (2016) where patients expressed the importance of being treated in accordance to social rules and with openness and acceptance. Not being able to talk openly about suicidality and underlying feeling increased anxiety, and the patients perceived themselves as a burden. Vandewalle et al. (2020) state that the sense of being cared for and acknowledged is pivotal for patients in a suicidal process. When patients were met with compassion and understanding, it challenged the perception of being a burden or that no one cared for them.

The present findings show that there is an ambivalence between living and dying as the patients navigate themselves and question what value they hold. This is in line with Hagen et al. (2020) where patients in a suicidal process describe being in a limbo, in a between phase where the outcome is uncertain. There is an uncertainty related to who one is as a person but also of who one can become. Suicidality seems to be relational and dialogical, and the interaction with clinicians impacted on this ambivalence and whether another future than suicide was conceivable.

Altogether, the present findings point to the important role that ambulance service could play in suicide prevention, since ambulance clinicians encounter patients in all phases of a suicidal process. Emergency departments have previously been described as an important context for suicide prevention (Ross et al., 2023). However, ambulance clinicians could have a suicide preventive role as well, both in enabling discourse of suicidal ideation and in the care of patients who have self-harmed. In the ambulance service, patients with risk factors of suicide are frequently present, for instance patients of old age and comorbidity (Erlangsen et al., 2015), but also patients with mental illness and self-harm behaviour (Duncan et al., 2019).

## Methodological considerations and study limitations

It could be considered a limitation that participants were only recruited from psychiatric clinics, as previous research shows that most patients do not have contact with psychiatry prior to suicide (Luoma et al., 2002). Psychiatric conditions are risk factors for suicide, yet most patients with psychiatric conditions do not die from suicide (Fazel & Runeson, 2020). Suicidality has been described as a parallel process to mental illness (Petrov, 2013), and the participants shared their experiences from the aspect of being in a suicidal process and encountering ambulance clinicians. Most of the situations described by the participants involved the observable phase of a suicidal process, such as suicide attempts. Ambulance clinicians are more likely to encounter patients in the non-observable phase of the suicidal process as this phase is more common (Wasserman, 2016), and this could be a limitation to this study. Yet, given that human interaction is pivotal for mental health and recovery, it could be assumed that the way ambulance clinicians relate to patients matters also in phases where suicidality is not observable by others. Another limitation could be that persons with conditions such as depression were not capable of participating but could still have had important experiences that would have enriched the findings. Also, there were no elderly persons among the participants which could limit the findings. Furthermore, it is possible that persons choose not to participate if they considered that the encounter lacked meaning. This could mean that the findings are overvalued due to the participation of those to whom it had meaning. At the same time, the vastly different experiences described in the findings, underlined by the quotations, could be considered a strength. There were rather few participants in this study, however their stories were rich and often included several experiences. Having follow-up interviews also enabled going even deeper in the meanings of the encounter.

Interviews enabled a deeper understanding of the phenomena in line with phenomenological hermeneutics (Lindseth & Norberg, 2004). However, interviews can be wearisome, and it is possible that potential participants declined participation of such reasons. Collecting data by surveys would have been an option to reach more participants but that would have limited the depth that was reached through interviews. The interviews were rather short, and this was partly because participants started to feel exhausted. Ending the first interviews and having follow-up interviews enabled the participants to continue sharing meanings and any reflections made during the time between the two interviews. Considering credibility, it must be considered that three out of four authors have experience from the ambulance service, and the fourth author has experience from psychiatric care. In phenomenological hermeneutics, there is a deliberate use of preunderstanding, for instance to guide which questions are to be asked (Lindseth & Norberg, 2004). At the same time, there is a risk that the analysis is negatively influenced by previous experience. This was handled through reoccurring discussions between all four authors in the analysis, and even though many interpretations are possible, not all interpretations are equally probable (Ricœur, 1976). The authors’ different pre-understanding is thus considered as a strength. The first author had a guiding role, as interviewer and verbatim transcriber, to ensure the most likely interpretation based on all authors’ discussions. The analysis was inductive and the decision to include The Tidal Model in the comprehensive understanding came after a dialogue among the authors and was suggested by the author with experience from psychiatric care. The Tidal Model and the theory of caring conversations were deemed to deepen the understanding of the structural analysis. Quotations from different participants have been provided to ensure confirmability of the findings. Regarding transferability, the variety of professions who work in the ambulance service around the world could limit the findings. However, even though the findings show the authority that ambulance clinicians uphold, the interpersonal relationship between patient and clinician points to something beyond the profession. The findings uncover an existential view of human interaction which strengthens the transferability to other settings and contexts.

## Conclusion

Patients in a suicidal process navigate themselves through the way ambulance clinicians communicate and interact with them. The encounter impacts on the patients’ ambivalence about living or dying, and it is in the clinicians’ power to consolidate the feeling of being a burden, or to instil hope of an endurable life. The encounter offers the possibility for the start of the patients’ journey of recovery, not only as a safe haven but also for the patients to reclaim their stories. Even if the encounter is short in terms of time, the brief togetherness could be an opportunity to be reminded of human relationship and important aspects of life. However, a start of recovery in the encounter is only possible if the ambulance clinicians listen attentively, are compassionate and show that they are trying to understand.

Further research is needed regarding ambulance and emergency care in a wider perspective of the patients’ journey of recovery.

## Implications

- Ambulance clinicians could play an important role in suicide prevention, both in enabling disclosure of suicidal ideation and in care after a suicide attempt.
- Competence development in suicidality and conversation skills could facilitate caring conversations with patients in a suicidal process.
- Through caring conversations, ambulance clinicians could support the patients in finding their own resource and value and thereby taking the first steps in the journey of recovery.
